# Positional Cloning Reveals Strain-Dependent Expression of *Trim16* to Alter Susceptibility to Bleomycin-Induced Pulmonary Fibrosis in Mice

**DOI:** 10.1371/journal.pgen.1003203

**Published:** 2013-01-17

**Authors:** Anguel N. Stefanov, Jessica Fox, Christina K. Haston

**Affiliations:** Meakins-Christie Laboratories and Department of Medicine, McGill University, Montreal, Canada; The Jackson Laboratory, United States of America

## Abstract

Pulmonary fibrosis is a disease of significant morbidity, with no effective therapeutics and an as yet incompletely defined genetic basis. The chemotherapeutic agent bleomycin induces pulmonary fibrosis in susceptible C57BL/6J mice but not in mice of the C3H/HeJ strain, and this differential strain response has been used in prior studies to map bleomycin-induced pulmonary fibrosis susceptibility loci named *Blmpf1* and *Blmpf2*. In this study we isolated the quantitative trait gene underlying *Blmpf2* initially by histologically phenotyping the bleomycin-induced lung disease of sublines of congenic mice to reduce the linkage region to 13 genes. Of these genes, *Trim16* was identified to have strain-dependent expression in the lung, which we determined was due to sequence variation in the promoter. Over-expression of *Trim16* by plasmid injection increased pulmonary fibrosis, and bronchoalveolar lavage levels of both interleukin 12/23-p40 and neutrophils, in bleomycin treated B6.C3H-*Blmpf2* subcongenic mice compared to subcongenic mice treated with bleomycin only, which follows the C57BL/6J versus C3H/HeJ strain difference in these traits. In summary we demonstrate that genetic variation in *Trim16* leads to its strain-dependent expression, which alters susceptibility to bleomycin-induced pulmonary fibrosis in mice.

## Author Summary

Genetic differences within the population influence an individual's susceptibility to the lung disease pulmonary fibrosis. As environmental factors also have a tremendous effect on the development of this disease, investigations in an animal model can reveal the genetic basis of this trait, under controlled circumstances. Starting from previous work that had identified a genomic region linked to fibrosis susceptibility in mice, we assayed the fibrosis response of lines of mice specifically bred to contain reduced portions of the original genetic interval, and we narrowed our study to 13 genes. Genetic evaluation pointed to the gene *Trim16* as a prime candidate for affecting fibrosis, and we identified genetic variations to alter its transcription. Our functional studies showed that *Trim16* injected into the specifically bred, and bleomcyin-treated, mice significantly increased their pulmonary fibrosis levels. Further evaluation of the mice showed the increase to be associated with known enhancers of fibrosis, neutrophils and interleukin12/23-p40. This study shows that genetic variation in *Trim16* affects both the lung tissue inflammatory response and the development of pulmonary fibrosis in mice and thus provides a novel pathway to fibrosis development for subsequent clinical investigation.

## Introduction

The pathology of pulmonary fibrosis features excessive deposition of extracellular matrix in the lung interstitium which can occur as the result of known (environmental, therapeutic) exposures, or idiopathically, and can result in impaired lung function and, ultimately, respiratory failure. This devastating disease has an estimated annual incidence of approximately 10 cases per 100000 members of general population [1] and an associated mortality of 50% at 3 years post diagnosis [2]. The mechanisms through which pulmonary fibrosis develops are incompletely understood but likely involve dysregulated repair of alveolar epithelial cell injury [2] which may be influenced by a tissue inflammatory response [3], [4].

Evidence for a genetic component to pulmonary fibrosis development includes heritability of the trait in family studies [2] and recent findings from linkage and association investigations. Regarding the latter, variation in particular genes encoding ELMO domain containing 2 [5], telomerase and surfactant proteins has been associated with the trait [6], and combined investigations of linkage in affected families, and of candidate gene assessment by association and pulmonary expression, have associated variants in the mucin gene *MUC5B* with pulmonary fibrosis susceptibility [7], [8]. Only a fraction of the genetic variation which contributes to this trait has been accounted for [6], however, and given the limits of case control association studies [9], which can include gene-environment effects, complementary investigations into the genetic basis of this disease in an animal model have been undertaken.

Investigations of mouse models wherein the fibrosis phenotype can be recapitulated in subjects controlled for age, genetic background and environmental exposure, can be completed to isolate candidate genetic variation contributing to the lung response phenotype. Specifically, the lung phenotype of C57BL/6J (B6) mice, following a 7day subcutaneous dose of bleomycin, consists of an alveolar inflammatory cell infiltrate with subpleural regions of fibrosis; a pathology that has been described for clinical cases of idiopathic pulmonary fibrosis [10]. This bleomycin delivery method, developed by Harrison *et al.* [11], and used by us [12], [13] has also been found to produce more fibrosis in the lung, and a fibrotic phenotype more closely resembling idiopathic pulmonary fibrosis, than the more commonly used experimental method of intratracheal drug delivery [14], [15]. In contrast to B6, mice of the C3Hf/KAM or C3H/HeJ strains develop minimal fibrosis following bleomycin treatment [13], [16] and this phenotypic difference was used to map loci of bleomycin-induced pulmonary fibrosis named *Blmpf1* on Chr 17 and *Blmpf2* on Chr 11 [17]. From the linkage study it was established that the inheritance of B6 alleles at either of *Blmpf1* and *Blmpf2* increased the fibrosis phenotype of B6×C3H F2 mice. The protective influence of Chromosome 11 C3H alleles on the fibrosis phenotype has been confirmed as bleomycin-challenged B6.11^C3H^ consomic mice were shown to develop significantly less pulmonary fibrosis than the levels in B6 mice [17].

Herein we generate and phenotype *Blmpf2* congenic and subcongenic mice to show *Trim16* to be the quantitative trait gene underlying *Blmpf2*.

## Results

### Reduction of the *Blmpf2* linkage region in subcongenic mice

**Figure 1:**
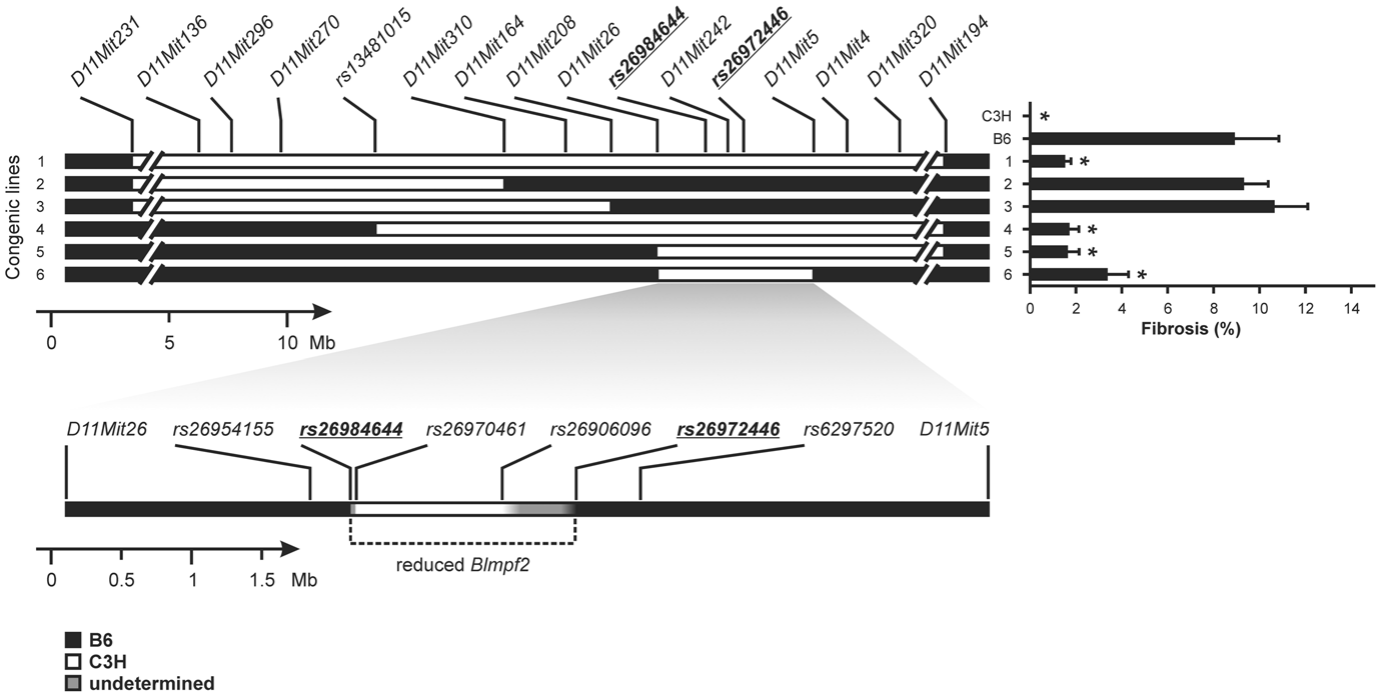
Bleomycin-induced lung phenotype of *Blmpf2* subcongenic mice. The mice were treated with bleomycin by mini-osmotic pump and euthanized 42 days later. The percentage of the lung with fibrosis was determined from image analysis of histological sections and the mean ± SEM of 10–19 bleomycin-treated mice for each subcongenic line, and for the parental strains, is given. * indicates a significant difference in fibrosis from B6 mice, p<0.05. Genotypes [C3H alleles (white box); B6 alleles (black box)] were determined with microsatellite and SNP markers; genotypes of line 6 are expanded at the bottom of the figure.

We initially confirmed *Blmpf2* as a locus altering fibrosis susceptibility by generating and phenotyping mice in which a 29.8 Mb fragment of Chromosome 11, encompassed by the *D11Mit136* and *D11Mit320* markers, from the lung fibrosis-resistant C3H strain, was bred onto the genetic background of lung fibrosis susceptible C57BL/6J mice, by repeated backcrossing. As shown in Figure 1, B6.*Blmpf2* ^C3H^ congenic mice (line 1) developed significantly less fibrosis (p = 0.014) in response to bleomycin treatment than B6 mice, but a level exceeding that of C3H/HeJ mice, (p = 0.002) which is likely due to the presence of B6 alleles at the *Blmpf1* locus in the congenic mice.

To narrow the *Blmpf2* linkage interval and thus to reduce the number of positional candidate genes contained we created and phenotyped a panel of *Blmpf2* subcongenic mice. As shown in Figure 1, mice (named B6.*Blmpf2* ^C3H^ subcongenic mice, line 6 in Figure 1) with C3H alleles from, maximally, marker *rs26984644* Chr11:62382506 to *rs26972446* Chr11:64009126 developed the same level of pulmonary fibrosis as the congenic strain (p = 0.95), thus mapping the *Blmpf2* fibrosis susceptibility gene to a region of 1.6 Mbp.

### Expression and genetic analysis of candidate genes

**Figure 2:**
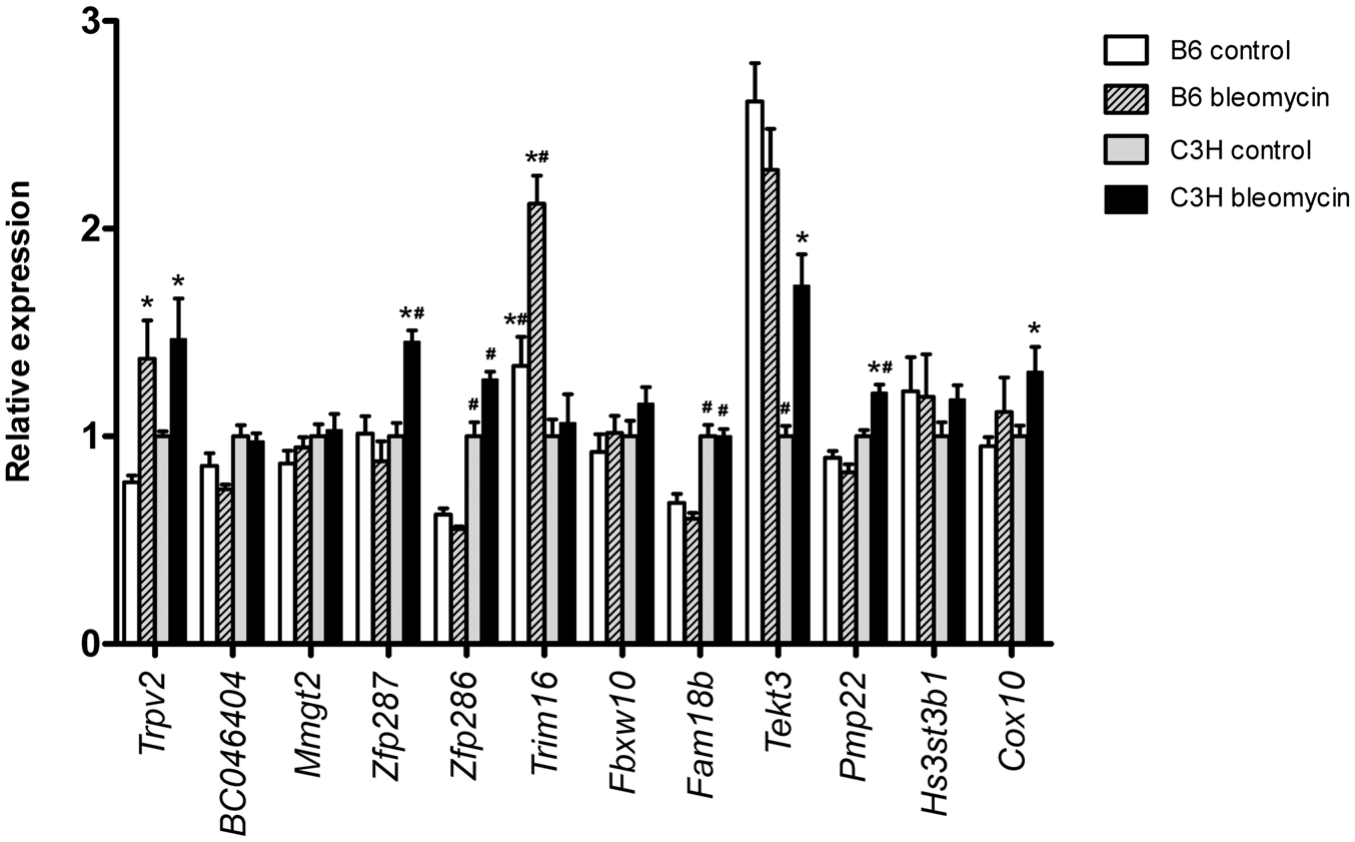
Pulmonary expression of reduced region *Blmpf2* genes. Real-time quantitative PCR of genes mapping to the reduced *Blmpf2* region prior to (day 0: non-treated) and following bleomycin treatment (day 42) in the lungs of B6 and C3H mice. Gene expression was normalized to the Ataxin10 reference gene and is presented relative to the level in untreated C3H mice. Mean ±SEM of 5 per group. * indicates a significant difference in expression in lungs of bleomycin-treated mice relative to untreated controls, p<0.05; ^#^ indicates a significant difference in expression by strain, p<0.05.

The reduced 1.6 Mbp region of *Blmpf2* contains 13 annotated genes (Genome Reference Consortium: GRCm38; MGI and NCBI gene annotation listed in Table S1). To assess each of the positional candidate genes as potentially contributing to the fibrosis phenotype we measured their expression levels in the lungs of both untreated B6 and C3H mice and following exposure to bleomycin and reviewed documented strain-dependent DNA sequence variation. As shown in Figure 2 one gene, tripartite motif-containing 16 (*Trim16*), of strain dependent expression, was significantly increased in expression in the lungs of bleomycin-treated B6 mice, compared to the levels in both untreated B6 mice and C3H mice exposed to bleomycin. The gene expression levels agree with data we had previously reported from a microarray study of the B6 and C3H response to bleomycin (GEO record GDS1492; ref 13), for the 7 linkage region genes included in the array. The expression of *Cdrt4* was not detected in either strain. We also assessed the level of expression of each of the positional candidate genes, except *Cdrt4*, in the lungs of B6.*Blmpf2* ^C3H^ subcongenic mice (Figure S1). The majority of genes exhibited expression levels similar to those detected in the lungs from C3H mice, although the expression of *Tekt3* was similar to that in the lungs of B6 mice, suggesting trans regulation for this gene. Finally, the expression of *Trpv2* and *Zfp287* in the lung tissue from B6.*Blmpf2* ^C3H^ subcongenic mice differed from that measured in either inbred strain.

Each of the positional candidate genes was reviewed using data from the dbSNP 128 SNP and Sanger databases to identify those genes having coding non synonymous B6/C3H SNP's as these variations could potentially contribute to the fibrosis phenotype of B6 mice and four genes (*Zfp286*, *Zfp287*, *Trim16* and *Fam18b*) met this criterion. By *in silico* analysis [18], [19] the majority of the coding non synonymous B6/C3H variations were deemed tolerated or benign.

From the gene expression and SNP analysis, and based on data demonstrating the encoded protein to affect the secretion of cytokines of relevance of fibrosis [20], *Trim16* was further investigated as a fibrosis susceptibility gene.

### B6/C3H sequence variation in *Trim16*

**Figure 3:**
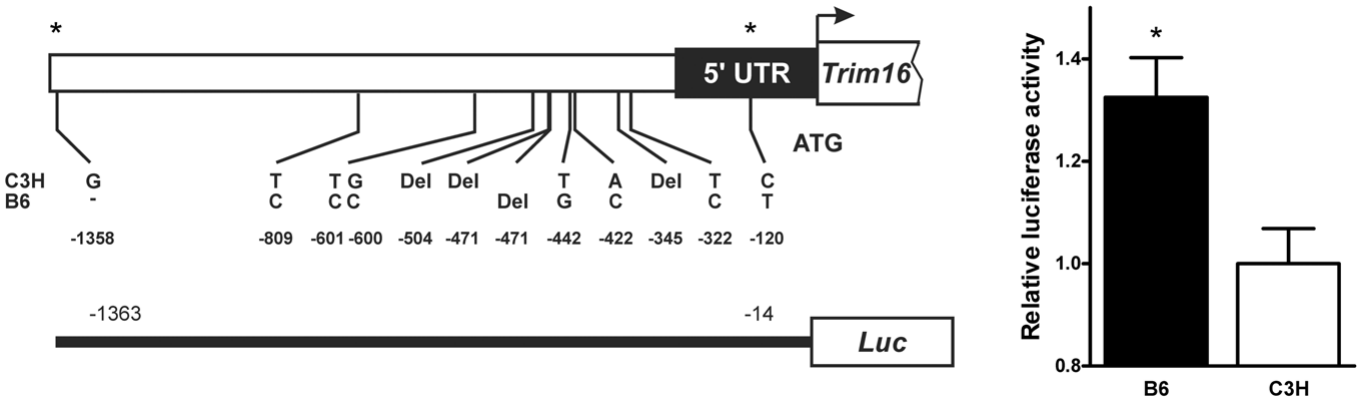
B6 and C3H *Trim16* promoter sequence variation alters transcription. B6/C3H sequence differences in the putative promoter region of *Trim16* (* indicates novel to Sanger, MGI). Allele specific promoter sequence alters the expression of a luciferase reporter vector transfected into the RAW 264.7 macrophage cell line.

Given the strain dependent expression of *Trim16*, we investigated the 5′ putative regulatory region for sequence differences which could create this differential expression. As shown in Figure 3 sequence differences in region 5′ to the transcription start site were documented, the majority of which verify data from the Sanger database [21]. Variations which are both evolutionarily conserved (see Figure S2) and which, based on data in PROMO, [22], [23] TRANSFAC [24] and MATCH [25], potentially alter transcription factor binding sites were revealed. To test the influence of this sequence on gene transcription, we completed a promoter assay and as shown in Figure 3, the B6 allele of the region 1.3 kbp upstream of the start site enhanced transcription relative to the C3H allele.

### Elevated *Trim16* expression increases fibrosis in B6.*Blmpf2* ^C3H^ subcongenic mice

**Figure 4:**
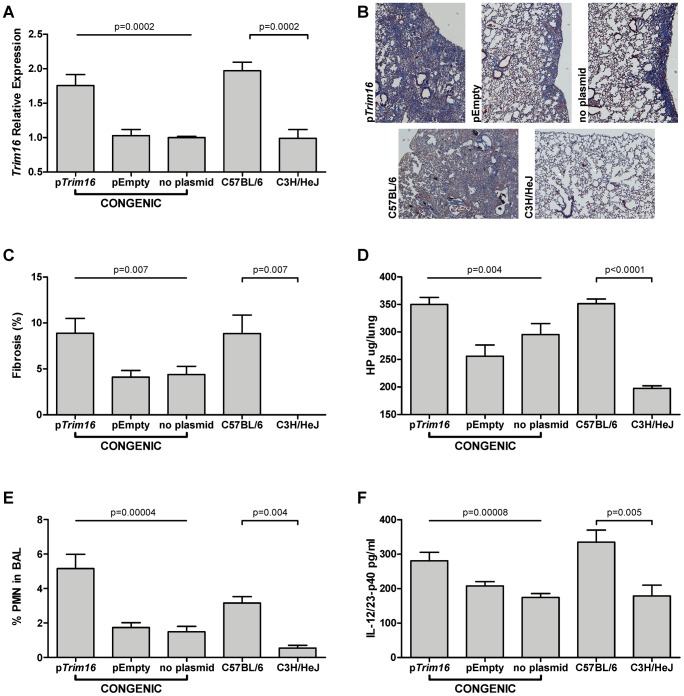
*Trim16* over-expression increases pulmonary fibrosis in *Blmpf2* subcongenic mice. Mice were treated with bleomycin as in Figure 1 and groups of *Blmpf2* subcongenic mice were treated with a *Trim16* expressing plasmid, an empty plasmid, or received no further treatment, 21 days after bleomycin treatment was initiated. The mice were euthanized at day 42. A. Right lung *Trim16* expression, by RT-PCR; n = 4–5 per group. B. histological sections of left lungs of congenic mice, top row and inbred strains, second row, Masson's trichrome stain, magnification of 100×. C. percent fibrosis in the left lung; n = 16–19 per group. D. total right lung hydroxyproline; n = 11–13 per group. E. Bronchoalveolar lavage neutrophil counts; n = 8–13 per group. F. Bronchoalveolar lavage supernatant interleukin-12/23p40 levels n = 8–13 per group. The average of each phenotype ± std. err. is given.

To investigate whether the altered expression of *Trim16* influences the fibrosis phenotype we treated B6.*Blmpf2* ^C3H^ subcongenic mice with bleomycin and injected a plasmid of *Trim16* in complex with the polyethylenimine (PEI) carrier [26], [27], at day 21 after the initiation of bleomycin treatment. The expression of *Trim16* in untreated subcongenic mice was similar to that of untreated C3H mice, (1.09 relative to the C3H level; p = 0.78) which is consistent with cis regulation. Intravenous injection of the *Trim16* plasmid increased expression of *Trim16* in the lungs of bleomycin-treated subcongenic mice, to B6 levels, as shown in Figure 4A.

B6.*Blmpf2* ^C3H^ subcongenic mice treated with bleomycin and the *Trim16* plasmid also developed significantly increased fibrosis, assessed both histologically and with hydroxyproline measures, compared to the levels in subcongenic mice receiving bleomycin alone or bleomycin and a control plasmid (Figure 4B–4D). The *Trim16* plasmid treatment resulted in B6.*Blmpf2* ^C3H^ subcongenic mice developing a level of fibrosis similar to that of B6 mice (p = 0.9), indicating a *Trim16* deficiency to have reduced the fibrosis development in subcongenic mice.

To elucidate how the fibrosis was enhanced in *Trim16* treated mice, the bronchoalveolar lavage fluid from each animal was scored for inflammatory cell types. Subcongenic mice treated with bleomycin and *Trim16* plasmid presented with neutrophil levels equal to those in bleomycin treated B6 mice (p = 0.6) and exceeding those of subcongenic mice receiving either of bleomycin alone or bleomycin and an empty vector, and the levels in bleomycin-treated C3H mice, as shown in Figure 4E.

Given that increased levels of the cytokine interleukin 12/23-p40, which is involved in neutrophil recruitment [28], can cause experimental pulmonary fibrosis [29], [30], we therefore assessed whether the increased fibrosis in the subcongenic mice was associated with a *Trim16* mediated enhancement of Il12/23-p40 secretion. We initially showed Il12/23-p40 levels to be relevant to fibrosis development in our model by measuring the level of this cytokine in the bronchoalveolar lavage of B6 and C3H mice following exposure to bleomycin. As shown in Figure 4F Il-12/23-p40 was increased to a greater extent in B6 than C3H mice, and in *Trim16* plasmid and bleomycin treated subcongenic mice compared to subcongenic challenged with bleomycin alone, thus elevated levels of *Trim16* in the bleomycin-treated lung affect the Il12/23-p40 pathway to fibrosis.

## Discussion

In previous studies the bleomycin-induced lung response of C57BL/6J mice, an established model of pulmonary fibrosis [31], was contrasted with that of C3H mice and used as the base of a linkage investigation mapping susceptibility to this lung disease to two loci [17]. In this work we moved from the mapped locus to the gene by demonstrating that overexpression of *Trim16* can contribute to pulmonary fibrosis development in mice.

Our approach to identify the *Blmpf2* quantitative trait gene included experiments in congenic and subcongenic mice, with candidate gene sequence variation and expression analyses, as in other positional cloning studies [32], [33]. Based on this genetic evidence the leading candidate of the genes mapping to the subcongenic interval was tripartite motif-containing 16, or *Trim16*, which encodes the protein Trim16. As Munding *et al.* [20] demonstrated that the secretion of the pro inflammatory cytokine interleukin-1β could be augmented *in vitro* by TRIM16, this gene was also a physiologically relevant candidate for fibrosis susceptibility owing to the inflammatory component of this trait.

To increase the expression of *Trim16* in the lungs of B6.*Blmpf2* ^C3H^ subcongenic mice to the levels in the fibrosis prone B6 strain we made use of a plasmid transfection system which had previously been shown to produce a high level of transfected gene expression in the lung without an increase in the tissue inflammatory response [26], [27], [34], [35]. With this system the elevated *Trim16* expression significantly increased the pulmonary fibrosis of recipient congenic mice. Further to elevated pulmonary fibrosis the increased *Trim16* expression produced higher levels of both Il12/23-p40 and neutrophils in the bronchoalveolar lavage of congenic mice. These findings are relevant as lavage neutrophilia has been associated with pulmonary fibrosis both experimentally [36], and clinically, where Kinder *et al.* [37] showed greater levels of neutrophils in the lavage to correlate with early mortality from pulmonary fibrosis.

Altered levels of the cytokine interleukin 12/23-p40 have also been implicated in pulmonary fibrosis pathology through studies in animal models. Specifically, Huaux *et al.* [29] showed the levels of interleukin 12-p40 to increase and to remain elevated in a model of experimental pulmonary fibrosis and for this cytokine subunit to decrease in abundance in resolving lung disease. In a subsequent study, Huaux *et al.* [30] demonstrated interleukin 12-p40 deficient mice to be protected from fibrosis development in response to challenge and, further, that supplementation with rIL-12p40 both restored the impaired pulmonary fibrotic response in these mice, and augmented the fibrosis in wild-type mice. Finally, Wilson *et al.* [36] reported bleomycin-induced pulmonary fibrosis and neutrophilia to be decreased in interleukin 12-p40 deficient mice.

Using a positional cloning strategy we have uncovered a novel gene of pulmonary fibrosis susceptibility in the mouse. Elevated levels of this gene, *Trim16*, increased both the fibrosis and a specific inflammatory phenotype in mice which supports the relevance of tissue inflammation to fibrosis development in this animal model [3]. Mechanistically, although Trim16 can interact with cytokines to increase their expression [20], and a genetic deficiency of related family member Trim21 has been shown to enhance Il12/23p-40 secretion [38], whether Trim16 directly or indirectly results in increased Il12/23-p40 remains to be determined. The finding that allele specific variation in *Trim16* can affect both fibrosis susceptibility and lavage neutrophilia could have important translational implications as both the disease phenotype and a subphenotype may be plausibly evaluated for clinical association; which, if confirmed would reveal a specific pathway for the development of this complex disease.

## Materials and Methods

### Ethics statement

All animals were treated and maintained under a protocol approved by the Animal Care Committee of McGill University, in accordance with guidelines set by the Canadian Council on Animal Use and Care.

### Mice

C57BL/6J and C3H/HeJ mice were purchased from Jackson Laboratories (Bar Harbor, ME) and housed in the Meakins Christie's animal facility. *Blmpf2* congenic mice were bred by intercrossing the strains, followed by backcrossing successive generations to B6 mice and selecting for C3H alleles in the linkage region of Chromosome 11, followed by intercrossing at the 10^th^ generation. To produce subcongenic mice the congenic mice were crossed to B6 mice, the resultant progeny were intercrossed and their offspring screened for recombination across the *Blmpf2* interval using microsatellite markers. Sister/brother progeny segregating the same recombinant intervals were intercrossed to generate strains harbouring homozygous subcongenic intervals. The position of the recombinant events was subsequently refined with SNP genotyping based on the Sanger database (http://www.sanger.ac.uk/resources/mouse/genomes/) and SNP detection performed using Taqman allelic discrimination assays for SNPs rs3023266, rs13481060 and rs6297520 and direct sequencing of amplified PCR products containing SNP sites.

### Pulmonary fibrosis phenotyping

Lung damage was elicited by challenging 8 to 10 week old mice with bleomycin sulphate dissolved in sodium chloride (Mayne Pharma, Canada). The mice were treated with 100–125 units/kg of bleomycin through mini-osmotic pumps (Alzet 2001, USA) implanted subcutaneously in the mouse's back as in prior studies [12], [13]. The pump was removed on day 8. The mice were euthanized six weeks later, bronchoalveolar lavage was completed and the lungs were preserved for histology, hydroxyproline measures or gene expression.

For histological analysis the left lungs were perfused with 10% neutral buffered formalin, embedded in paraffin blocks, sectioned and stained with Masson's Trichrome. The fibrosis area was quantified and compared with the surface of the entire lung to yield a percentage of fibrosis using the Image-Pro software (Media Cybernetics) as in prior studies [12], [13], [16]. The hydroxyproline content of the mouse lungs was determined using previously described standard methods [39]. For gene expression lung tissue was immediately homogenized in 2 ml TRI reagent (Sigma-Aldrich, USA) and expression was assessed with Applied Biosystem's assays on demand as in previous studies [40], [41].

### Bronchoalveolar lavage fluid analysis

The lungs were lavaged with one mL of PBS, the lavage fluid was centrifuged (302 g for 10 minutes at 4°C) and the supernatant was removed and stored at −85°C. The cellular pellet was resuspended in 0.25 mL PBS. Inflammatory cell counts were performed (400× magnification) on cytocentrifuged cells (214.2 g for three minutes), after staining with a hematoxylin-eosin kit (Hema-3 Stain Set by Protocol).

### Sequencing

A 1368 bp region containing the putative *Trim16* promoter from strains C57BL/6J and C3H/HeJ was amplified and cloned in plasmid pJet1.2/blunt (Thermo Fisher Scientific). Inserts were sequenced and compared to the mouse genome assembly of the UCSC database (http://genome.ucsc.edu) and to that in the Sanger database to evaluate strain dependent polymorphisms.

### Transcription factor binding site analysis

Putative transcription factor binding sites located within the 1.4 kb of the promoter region were identified *in silico* using the PROMO program [22], [23] and TFsearch (v.1.3) of the TRANSFAC database [24] with a focus on murine specific transcription factors and transcription factor binding sites. The sequence was also analyzed with the MATCH public 1.0 algorithm by Biobase GmbH [24] using vertebrate weight matrices and a minimum false negative profile.

In order to determine evolutionary conservation of promoter region B6/C3H SNPs or deletions we used the Multiz Alignment & Conservation algorithm of the UCSC genome browser (http://genome.ucsc.edu; [42]). The graphic output was created using CodonCode Aligner (v. 3.7.1, Codon Code Corporation).

### Promoter cloning and transcription assay

A 1368 bp fragment (−1372 to −4 bp) was amplified from strains C57BL/6J and C3H/HeJ by PCR and cloned into the KpnI/HindIII sites of vector pGL3-Basic (Promega, Madison, WI). Sequencing of the inserts was completed to confirm allelic specificity. RAW246.7 macrophage cells were co-transfected with the reporter vector, or empty pGL3-basic, and pRL-SV40 (Promega), which was used as an internal control for transfection efficiency. Twenty four hours after transfection, cells were assayed for both firefly and renilla luciferase activity using the Dual-Luciferase Reporter Assay System (Promega, Madison, WI).

### Plasmid delivery to mouse lung

A plasmid of the full length B6 mouse *Trim16* cDNA with the 252 bp 5′ UTR and 3′ UTR (accession NM_053169), cloned into the EcoRI – NotI sites of expression vector pDream2.1/MCS, and an empty modified pDream2.1/MCS vector, were obtained from Genscript (GenScript, USA). Prior to cloning the vector was modified by removing an 18 nt Flag tag sequence to avoid possible interference with the expressed protein structure. Plasmid insert specificity and orientation were confirmed by sequencing. The cationic linear polyethylenimine *in vivo*-jetPEI (Polyplus-transfection, France; MJS Biolynx, Canada) was used for plasmid transfection to the mouse lung according to the manufacturer's instructions. The PEI (6.5 µl) and *Trim16* plasmid (40 µg) complex was resuspended in a sterile 5% glucose solution (400 uL total) and injected into the tail vein of mice.

### ELISA

The level of Il-12/23-p40 in the bronchoalveolar lavage supernatant was assayed with a murine Il-12/23(p40) ELISA kit (eBioscience, USA) according to the manufacturer's instructions.

### Statistics

Phenotypic differences between groups were assessed with Student's t test and among groups of congenic mice receiving distinct treatments, as in Figure 4, by an analysis of variance followed by Tukey post hoc tests.

## Supporting Information

Figure S1 Pulmonary expression of reduced region *Blmpf2* genes in *Blmpf2* subcongenic mice. Real-time quantitative PCR of genes mapping to the reduced *Blmpf2* region prior to (day 0: non-treated) and following bleomycin treatment (day 42) in the lungs of *Blmpf2* subcongenic mice, relative to that of the parental B6 and C3H mice. Gene expression was normalized to the Ataxin10 reference gene and is presented relative to the level in untreated C3H mice. Mean ±SEM of 5 per group. * indicates a significant difference in expression in lungs of bleomycin-treated mice relative to untreated controls, p<0.05; ^#^ indicates a significant difference in expression to B6 mice, p<0.05.
(TIF)

Figure S2 B6/C3H variation in *Trim16* promoter sequence is evolutionarily conserved. The Multiz Alignment & Conservation algorithm was used to evaluate evolutionary conservation of polymorphisms within the 1368 bp region upstream *Trim16* ATG. Four polymorphisms were found to have different degrees of conservation among the species. A. SNP rs26955306 at position −422; B. deletion between SNP −422 and −322; C. SNP rs49831756 at position −322 and D. novel SNP at position −120.
(TIF)

Table S1 NCBI and MGI identifiers of genes and loci described in this study.
(DOC)
