# Correction: Positional Cloning Reveals Strain-Dependent Expression of *Trim16* to Alter Susceptibility to Bleomycin-Induced Pulmonary Fibrosis in Mice

**DOI:** 10.1371/annotation/f184e509-5c53-48da-b94a-b7517f591b3e

**Published:** 2013-10-22

**Authors:** Anguel N. Stefanov, Jessica Fox, François Depault, Christina K. Haston

François Depault was not included in the author byline. He is listed as the third author and affiliated with Meakins-Christie Laboratories and Department of Human Genetics, McGill University, Montreal, Canada.

The corrected citation is Stefanov AN, Fox J, Depault F, Haston CK (2013) Positional Cloning Reveals Strain-Dependent Expression of *Trim16* to Alter Susceptibility to Bleomycin-Induced Pulmonary Fibrosis in Mice. PLoS Genet 9(1): e1003203. doi:10.1371/journal.pgen.1003203

The corrected author contributions are: Conceived and designed the experiments: CKH ANS. Performed the experiments: ANS JF. Analyzed the data: ANS FD CKH. Wrote the paper: CKH. 

