# Supplementary figures and images for: Positional Cloning Reveals Strain-Dependent Expression of Trim16 to Alter Susceptibility to Bleomycin-Induced Pulmonary Fibrosis in Mice
Source: PLoS Genet. 2013 Jan 17;9(1):e1003203. doi: 10.1371/journal.pgen.1003203 (PMC3547790; doi:10.1371/journal.pgen.1003203)

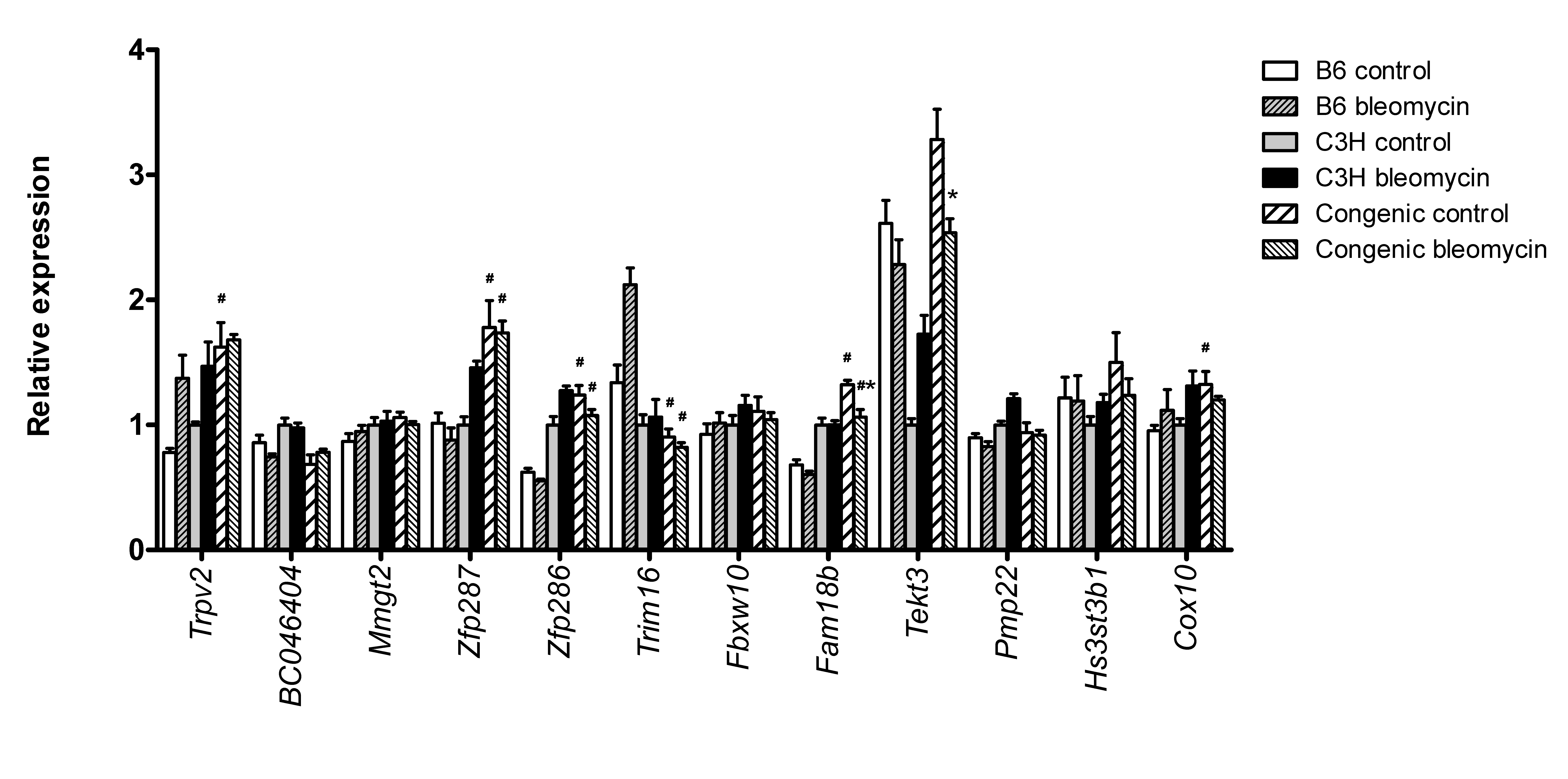

Supplement: Figure S1 — Pulmonary expression of reduced region Blmpf2 genes in Blmpf2 subcongenic mice. Real-time quantitative PCR of genes mapping to the reduced Blmpf2 region prior to (day 0: non-treated) and following bleomycin treatment (day 42) in the lungs of Blmpf2 subcongenic mice, relative to that of the parental B6 and C3H mice. Gene expression was normalized to the Ataxin10 reference gene and is presented relative to the level in untreated C3H mice. Mean ±SEM of 5 per group. * indicates a significant difference in expression in lungs of bleomycin-treated mice relative to untreated controls, p<0.05; # indicates a significant difference in expression to B6 mice, p<0.05. (TIF) [file pgen.1003203.s001.tif]

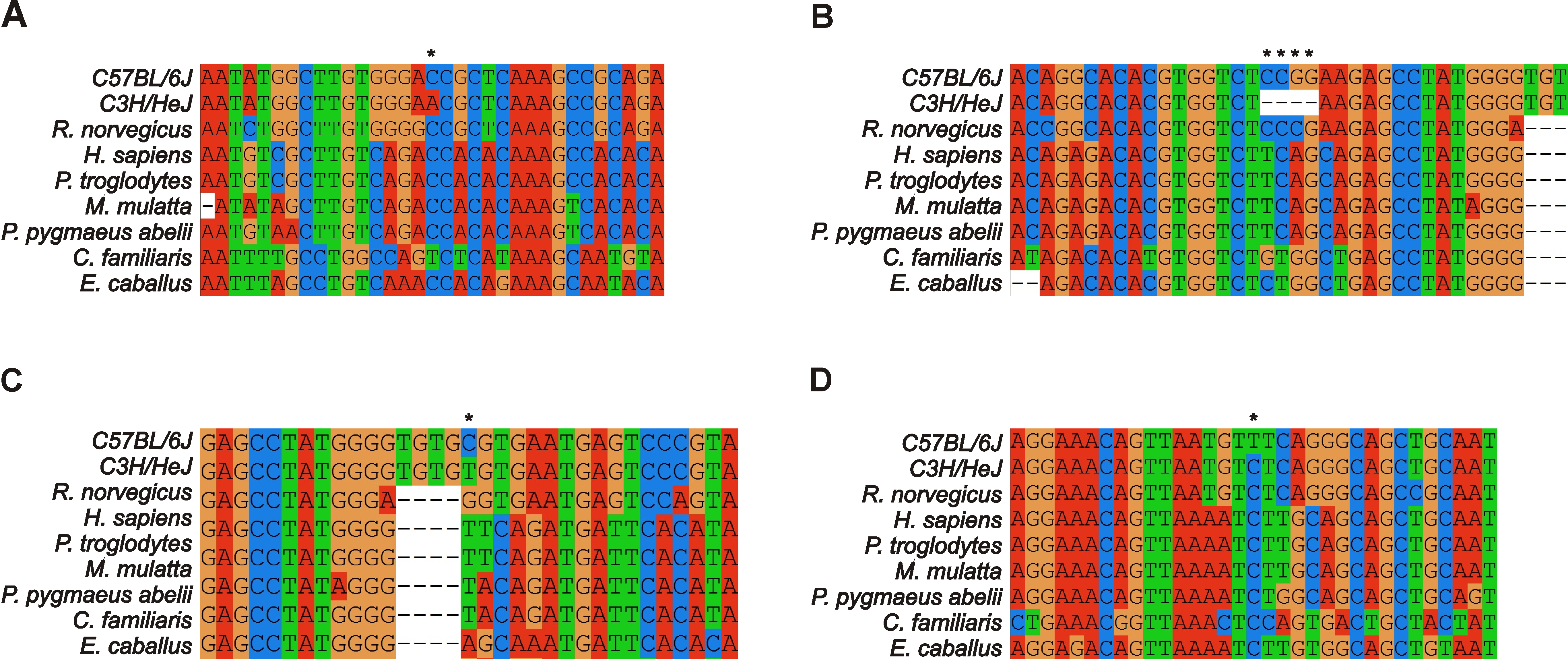

Supplement: Figure S2 — B6/C3H variation in Trim16 promoter sequence is evolutionarily conserved. The Multiz Alignment & Conservation algorithm was used to evaluate evolutionary conservation of polymorphisms within the 1368 bp region upstream Trim16 ATG. Four polymorphisms were found to have different degrees of conservation among the species. A. SNP rs26955306 at position −422; B. deletion between SNP −422 and −322; C. SNP rs49831756 at position −322 and D. novel SNP at position −120. (TIF) [file pgen.1003203.s002.tif]
