# Supplementary material for: Positional Cloning Reveals Strain-Dependent Expression of Trim16 to Alter Susceptibility to Bleomycin-Induced Pulmonary Fibrosis in Mice
Source: PLoS Genet. 2013 Jan 17;9(1):e1003203. doi: 10.1371/journal.pgen.1003203 (PMC3547790; doi:10.1371/journal.pgen.1003203)
Supplement: Table S1 — NCBI and MGI identifiers of genes and loci described in this study. (DOC) [file pgen.1003203.s003.doc]

Supplemental Table 1

|  | NCBI Gene | MGI |
| --- | --- | --- |
| *Blmpf1* | 492880 | 2668887 |
| *Blmpf2* | 492881 | 2668888 |
| *Trim16* | 94092 | 2137356 |
| *Cdrt4* | 66338 | 1913588 |
| *Zfp286* | 192651 | 2384758 |
| *Zfp287* | 170740 | 2176561 |
| *Fam18b* | 67510 | 1914760 |
| *Trpv2* | 22368 | 1341836 |
| *BC046404* | 192976 | 2682293 |
| *Mmgt2* | 216829 | 2448491 |
| *Fbxw10* | 213980 | 3052463 |
| *Tekt3* | 71062 | 1918312 |
| *Pmp22* | 18858 | 97631 |
| *Hs3st3b1* | 54710 | 1333853 |
| *Cox10* | 70383 | 1917633 |

NCBI and MGI identifiers of genes and loci described in this study.
